# Photosynthetic Acclimation of *Symbiodinium in hospite* Depends on Vertical Position in the Tissue of the Scleractinian Coral *Montastrea curta*

**DOI:** 10.3389/fmicb.2016.00230

**Published:** 2016-02-26

**Authors:** Mads Lichtenberg, Anthony W. D. Larkum, Michael Kühl

**Affiliations:** ^1^Marine Biological Section, Department of Biology, University of CopenhagenHelsingør, Denmark; ^2^Plant Functional Biology and Climate Change Cluster (C3), University of Technology SydneySydney, NSW, Australia

**Keywords:** canopy effects, chlorophyll fluorescence, microsensors, photosynthesis, photo-acclimation, scalar irradiance, zooxanthellae

## Abstract

Coral photophysiology has been studied intensively from the colony scale down to the scale of single fluorescent pigment granules as light is one of the key determinants for coral health. We studied the photophysiology of the oral and aboral symbiont band of scleractinian coral *Montastrea curta* to investigate if different acclimation to light exist *in hospite* on a polyp scale. By combined use of electrochemical and fiber-optic microsensors for O_2_, scalar irradiance and variable chlorophyll fluorescence, we could characterize the physical and chemical microenvironment experienced by the symbionts and, for the first time, estimate effective quantum yields of PSII photochemistry and rates of electron transport at the position of the zooxanthellae corrected for the in-tissue gradient of scalar irradiance. The oral- and aboral *Symbiodinium* layers received ∼71% and ∼33% of surface scalar irradiance, respectively, and the two symbiont layers experience considerable differences in light exposure. Rates of gross photosynthesis did not differ markedly between the oral- and aboral layer and curves of PSII electron transport rates corrected for scalar irradiance *in hospite*, showed that the light use efficiency under sub-saturating light conditions were similar between the two layers. However, the aboral *Symbiodinium* band did not experience photosynthetic saturation, even at the highest investigated irradiance where the oral layer was clearly saturated. We thus found a different light acclimation response for the oral and aboral symbiont bands *in hospite*, and discuss whether such response could be shaped by spectral shifts caused by tissue gradients of scalar irradiance. Based on our experimental finding, combined with previous knowledge, we present a conceptual model on the photophysiology of *Symbiodinium* residing inside living coral tissue under natural gradients of light and chemical parameters.

## Introduction

Coral reefs form one of the most diverse and productive ecosystems on Earth. The high productivity relies on the relationship between the endosymbiotic zooxanthellae (dinoflagellates in the genus *Symbiodinium*) and the coral host (Muscatine and Porter, 1977). The endosymbiont gains protection and nutrients, while the coral host relies on the energy supplied as carbohydrates by its phototrophic partner (Muscatine and Porter, 1977; Edmunds and Davies, 1986, 1989). Symbiont-bearing corals are limited to habitats with appropriate light conditions, but inhabit a wide span of light-exposed habitats ranging from shallow reef flats where mid-day solar irradiance reaches >2000 μmol photons m^-2^ s^-1^ to shaded caves (Anthony and Hoegh-Guldberg, 2003) and >150 m deep waters (Bridge et al., 2013) in virtual darkness. Colonization of such a wide range of habitats is facilitated by the ability of corals to modulate and optimize their tissue light environment and thereby the light exposure of the zooxanthellae. The regulation of internal light field serves to either filter out excess light that can be harmful to the algae, or to increase the photon flux reaching the algae in sun-exposed or shaded environments, respectively. There are several mechanisms by which the coral host optimizes the light environment for its endosymbionts, e.g., by (i) screening out harmful UV-radiation by chromoproteins (Smith et al., 2013) or other fluorescent host pigments (Salih et al., 2000), (ii) host pigment conversion of short-wave radiation to longer wavelengths, which are more efficient for photosynthetic conversion (Schlichter and Fricke, 1990; Gilmore et al., 2003), or (iii) increasing the internal photon flux density in the tissue by scattering and skeleton reflection (Enriquez et al., 2005; Wangpraseurt et al., 2014a). On a larger scale, Anthony et al. (2005) investigated irradiance levels inside foliaceous (leaf-like) corals and found that structural elements (colony plates) can regulate the light regime toward the maximum sub-saturation irradiance (*E*_k,max_). The light field in contrasting colony growth forms (branching vs. massive) has been shown to be the same at the level of the endosymbionts, despite different surface light environments (Kaniewska et al., 2011). In addition, corals with different tissue configuration (relaxed vs. contracted) show different light microclimates (Wangpraseurt et al., 2014a), and tissue plasticity may thus be very important for regulating the internal light regime toward the optimal conditions for coral endosymbionts.

Besides host-induced regulation of the light microclimate, the zooxanthellae can employ different strategies to regulate photon absorption, e.g., by regulating the concentration of light harvesting pigments and photoprotective pigments, and it has been shown that photoprotection can be achieved by varying the PSII antennae size, i.e., the functional absorption cross-section of PSII (Hill and Ralph, 2006; Hill et al., 2012).

Normally, shade adapted corals display high light use efficiencies but low maximal photosynthesis rates. This strategy involves employing a higher amount of light harvesting pigments and thus a greater absorption cross-section (Dubinsky et al., 1984), while the opposite is the case for corals adapted to high irradiance that often appear more transparent due to downregulation of light harvesting pigments and organization of chloroplasts to minimize light capture (Dubinsky et al., 1984). In shade, the photochemical conversion is limited by the supply of photons to the photosystems, whereas in full sunlight the enzymatic processes limit the energy transformation and as a result the coral is left with a surplus of photons. This surplus energy can be dissipated via non-photochemical quenching processes in order to avoid photoinhibition (Brown et al., 1999; Gorbunov et al., 2001; Cooper et al., 2011).

Reef-building corals have developed a number of strategies to succeed in highly variable light environments. Clearly one passive option is to adapt the light harvesting pigments to the average incident radiation (Ramus et al., 1977). At the same time, the zooxanthellae have the capacity to entrain non-photochemical quenching mechanisms to avoid high levels of photoinhibition when light levels become high (Brown et al., 1999) and to entrain the water-water cycle (Mehler ascorbate peroxidase pathway (Roberty et al., 2014). Furthermore, the coral host can modulate the light scattering in the coral tissue by (i) modifying the calcium carbonate skeleton (Marcelino et al., 2013) enhancing light absorption (Enriquez et al., 2005), and (ii) by modulating scattering in the tissue due to contraction and expansion strongly affecting intra tissue light levels (Wangpraseurt et al., 2014a). The coral host can also regulate the number of zooxanthellae engulfed in the endodermal cells as a response to irradiance (Stimson, 1997). Finally, corals produce a range of fluorescent protein-like pigments (FP), which can change the optical properties of the coral tissue in a number of ways. For instance, by (i) changing the scattering properties (Salih et al., 2000; Lyndby et al., submitted), (ii) by changing light quality within the host tissue (Salih et al., 2000), and (iii) by converting short wave radiation to longer wave radiation (Schlichter et al., 1986; Gilmore et al., 2003). While there is some evidence for all mentioned mechanisms for modifying light-harvesting in corals there is much work left to be done to fully understand the subtle processes involved and how they regulate *Symbiodinium* photosynthesis.

In this study we explored the photophysiology of *Symbiodinium in hospite* in the tissue of the massive scleractinian coral *M. curta* by the combined use of electrochemical and fiber optic microsensors. Electrochemical microsensors for O_2_ are important tools to unravel biogeochemical processes in, e.g., marine sediments (Revsbech and Jørgensen, 1983), microbial mats from extreme environments (Revsbech and Ward, 1984), aquatic macrophytes (Spilling et al., 2010; Brodersen et al., 2015; Lichtenberg and Kühl, 2015) and corals (Kühl et al., 1995). Combined with fiber-optic probes for either field radiance (directional photon flux) or scalar irradiance (integrated total photon flux) photosynthetic performance can be investigated in high spatial resolution inside phototrophic tissues or communities (Kühl et al., 1996; Brodersen et al., 2014). Measurements of variable chlorophyll fluorescence, using the saturation pulse method with pulse-amplitude-modulated (PAM) fluorometers (Schreiber, 2004), have become increasingly popular to assess photosynthetic performance in aquatic systems. However, only the micro fiber-based PAM system (Schreiber et al., 1996) allows high spatial resolution, intra-tissue measurements of photosynthetic parameters, by either applying actinic light through the fiber to assess potential quantum yields or by applying actinic light externally to measure effective quantum yields as a function of the gradient of light seen by the photosynthetic unit.

The symbiotic algae reside in the gastrodermal tissue and are thus spatially separated by the gastrovascular cavity. This separation means that oral and aboral symbiont layers can experience differences in light quantity and spectral composition due to absorption and scattering of light in the tissue (Wangpraseurt et al., 2012). Most studies of coral photosynthesis have ignored such symbiont stratification, but there is increasing evidence that such stratification can enable differential photoacclimation in the coral tissue (Wangpraseurt et al., 2015). Microniches enabling differential acclimation in different parts of coral tissue may be an important yet overlooked component governing efficient coral photosynthesis over a wide range of irradiance.

In this study, we investigated whether *Symbiodinium* cells located in oral and aboral tissue layers display similar light acclimation properties by characterizing intra-tissue light gradients, the oxic microenvironment and the depth distribution of photosynthetic rates and rates of PSII electron transport within the coral tissue. Such detailed information on photosynthetic performance of *Symbiodinium in hospite* could have important implications for the understanding of symbiont resilience against high-light stress.

## Materials and Methods

### Coral Samples

Coral samples were collected from shallow waters in Shark Bay, Heron Island (Capricornia Cays, Great Barrier Reef, Australia; 23°26′31′′S 151°55′30′′E). We selected the favid coral *M. curta* due to its suitability for microsensor studies owing to thick tissue and low mucus production (Wangpraseurt et al., 2012; Brodersen et al., 2014). After collection, coral fragments were transferred to a 50 L aquarium where they were maintained under a continuous flow of filtered seawater from the lagoon (temperature: ∼26°C; salinity: ∼36). The coral tank was located outdoors under a natural diurnal light cycle, but was shaded such that maximum midday photon irradiance (400–700 nm) was ∼500 μmol photons m^-2^ s^-1^. Three coral fragments were chosen for experiments. From these, polyps were randomly chosen across all coral fragments for individual measurements. However, at least one replicate was done on a polyp from each fragment; when *n* > 3, more than one polyp were measured on one of the fragments. Replicates across measurements were thus done on the polyp scale. The measurement points on individual polyps were chosen randomly on the oral disk tissue surrounding the polyp mouth.

### Experimental Setup

All measurements were conducted with a coral fragment placed in a custom-made black acrylic flow chamber (25 cm × 8 cm × 8 cm) supplied with aerated seawater (26°C; *S* = 36) at a flow velocity of ∼2 cm s^-1^ as provided by a water pump (Fluval U1, Rolf C. Hagen Ltd., England) in a 25 L aquarium with seawater that was continuously flushed with atmospheric air by an air pump (Sera Air 110 plus, Sera GmbH, Germany). We used fiber-optic and electrochemical microsensors to measure photon scalar irradiance, gross photosynthesis, O_2_ concentrations and variable chlorophyll fluorescence in vertical steps through the coral tissue (see details below and in Supplementary Figure S3). Positioning of the microsensors on the coral surface was done visually through a PC-interfaced USB-microscope (AM7013MZT Dino-Lite, AnMo Electronics Corporation, Taiwan). For measurements, the microsensors were mounted on a motorized micromanipulator (MU-1, PyroScience GmbH, Germany) controlled by a PC running dedicated software (ProFix, PyroScience GmbH, Germany).

### Light Measurements

Depth profiles of photon scalar irradiance in coral tissue were measured with fiber-optic scalar irradiance microprobes with a sphere diameter of ∼45 μm and an isotropic angular response (Rickelt et al., 2016). The scalar irradiance microprobe was connected to a fiber-optic spectrometer (USB2000+, Ocean Optics, USA) interfaced to a PC running spectral acquisition software (Spectra Suite, Ocean Optics, USA). Light was provided at a slight angle by a fiber optic tungsten halogen lamp (KL2500-LCD, Schott GmbH, Germany) equipped with a collimating lens. All measurements were performed in a dark room to avoid stray light. Profiles of photon scalar irradiance were measured in vertical steps of 0.1 mm from the surface of the coral tissue toward the skeleton, which was determined as the depth where the fiber of the microprobe bended slightly or retracted into the needle. To penetrate the coral tissue, a small incision in the tissue of the oral disk was carefully made with the tip of a hypodermic needle. During this procedure, the coral tissue contracted and corals were allowed 1–3 min to allow tissue relaxation before measurements of light microprofiles.

Incident light was quantified as the downwelling photon scalar irradiance from the fiber optic tungsten halogen lamp with the fiber optic microprobe positioned over a black, non-reflective light-well at a distance and position in the light field similar to the position of the coral surface; in a collimated light field, the downwelling irradiance and the downwelling scalar irradiance is identical (Kühl and Jørgensen, 1994). Absolute incident photon irradiance (PAR, 400–700 nm; in μmol photons m^-2^ s^-1^) was measured with a calibrated photon irradiance meter (ULM-500, Walz GmbH, Germany) equipped with a spherical sensor (US-SQS/L, Walz GmbH, Germany) positioned in the light-well at a distance similar to the position of the coral surface.

The acquired spectra were integrated over the spectral regions of interest, i.e., PAR (400–700 nm), and the integral was related to the absolute incident photon irradiance to obtain the amount of photosynthetic active radiation at each measuring depth expressed as fractions of incident photon scalar irradiance. The photon scalar irradiance attenuation coefficient, *K*_0_ (mm^-1^), was calculated as the slope of the natural logarithm transformed photon scalar irradiance plotted as a function of depth (Kühl, 2005). The spectral attenuation coefficient, *K*_0_(λ) (mm^-1^) was calculated as, **K*_0_(λ) = –ln[*E*_0_(λ)_1_/ *E*_0_(λ)_2_]/( z_2_ – z_1_)*, where *E*_0_(λ)_1_ and *E*_0_(λ)_2_ are the spectral scalar irradiances measured at depth *z*_1_ and *z*_2_, respectively (Kühl and Jørgensen, 1994; Kühl, 2005).

### Variable Chlorophyll Fluorescence

Microscale measurements of variable chlorophyll fluorescence using the saturation pulse method (Schreiber, 2004) were done with a sensitive fiber-optic fluorometer (Microfiber PAM, Waltz GmbH, Germany) (Schreiber et al., 1996; Ulstrup et al., 2006). The fiber-optic microprobe consisted of a single strand graded index multimode fiber cable (Radiall Inc., France) mounted in a syringe and needle with the measuring tip tapered and rounded to ∼30 μm at the light collecting end (Kühl, 2005) and connected to a sensitive pulse-amplitude modulated detector system at the other end via a fiber-optic beam splitter/coupler (see details in Schreiber et al., 1996). On the system end, one fiber branch of the splitter/coupler was connected to a LED light source providing measuring light and saturating pulses, while the other branch was connected to a sensitive photomultiplier-detector equipped with a long-pass filter to screen out the LED excitation light and only detect chlorophyll fluorescence. On the measuring side of the splitter/coupler, one branch was connected to the fluorescence microprobe, while the other branch was not used. To ensure good optical throughput, a small droplet of microscope immersion oil was added in the fiber-fiber connections. Fiber connections used the ST-connector standard.

The PAM control unit was connected to a LED ring (Ulstrup et al., 2006) providing known photon irradiance levels of red light (63, 93,142, 213, 303, 422, 695, and 1018 μmol photons m^-2^ s^-1^; peak emission: 666 nm; Supplementary Figure S1). The incident photon irradiance from the red LED ring at different settings was measured with a calibrated irradiance meter (ULM-500, Walz GmbH, Germany) equipped with a spherical sensor (US-SQS/L, Walz GmbH, Germany) positioned over a black light-well at a distance similar to the position of the coral surface. Data were collected using PC controlled data acquisition software (Win Control v. 2.08, Walz GmbH) that controlled the Microfiber PAM system.

We measured rapid light curves (RLC) and steady state light curves (LC) in the two spatially separated endosymbiont layers. Due to inter-polyp differences in the location of these endosymbiotic layers (**Figure 3**), we located the layers by monitoring the fluorescence yield signal. By slowly moving the optical fiber vertically through the tissue, the center of the layers was determined as the position showing the largest fluorescence yield. RLC’s (Ralph and Gademann, 2005) were measured with 10 s acclimation to increasing irradiance, while steady state LC were measured with 5 min acclimation to each of the increasing irradiance levels.

Local rates of relative photosystem II (PSII) related electron transport, rETR, were calculated from the effective quantum yield of PSII ignoring the absorption factor and the factor describing absorption by both photosystems; (Ralph et al., 2002) and by using the actual scalar irradiance measured locally instead of the incident irradiance (Lichtenberg and Kühl, 2015). Fitting of experimental rETR vs. scalar irradiance curves was done using an exponential function (Webb et al., 1974) yielding the maximum rate of electron transport through PSII (rETR_max_) and the light use efficiency (α; the initial slope of the rETR vs. scalar irradiance curve). Where data did not reach saturation, the initial slope (α) was linearly fitted below 150 μmol photons m^-2^ s^-1^ of scalar irradiance. The scalar irradiance at the onset of photosynthesis saturation, the so called *E*_k_ parameter, was calculated as *E_k_ = rETR_max_/α.* Curve fitting was done with the non-linear curve fitting functions of Origin 9.2 (OriginLab Corporation, Northampton, MA, USA).

### Measurements of O_2_ Concentration and Gross Photosynthesis

Profiles of O_2_ concentration and gross photosynthesis were measured in 0.1 mm vertical steps through the coral tissue using a Clark type O_2_ electrochemical microsensor (OX-25, Unisense, Denmark) (Revsbech, 1989) with a tip diameter of <25 μm, low stirring sensitivity (<1–2%) and a fast response time (*t*_90_ < 0.5 s). The microsensor was connected to a pA-meter (pA-2000, Unisense, Denmark) and signals were recorded on a strip-chart recorder (BD 12E, Kipp and Zonen B.V., Netherlands). The O_2_ microsensor was linearly calibrated from signal readings in air saturated seawater and in anoxic seawater (produced by addition of sodium sulfite to seawater at experimental temperature and salinity). Measurements were done at increasing photon irradiance (18, 63, 93, 213, 303, 695, and 1018 μmol photons m^-2^ s^-1^) of red light as provided by the red LED ring described above. Volumetric rates of gross photosynthesis (in nmol O_2_ cm^-3^ s^-1^) were calculated from the initial O_2_ depletion rate after a brief darkening following the light-dark shift method (Revsbech and Jørgensen, 1983). Depth integration of the volumetric rates measured throughout the tissue at each irradiance, yielded areal gross photosynthesis (in nmol O_2_ cm^-2^ s^-1^) vs. photon irradiance curves. Vertical profiles of O_2_ concentration were obtained during the gross photosynthesis measurements in each depth from the steady-state O_2_ concentration obtained in each measurement depth just before the brief darkening.

## Results

### Spectral Light Regime

**FIGURE 1:**
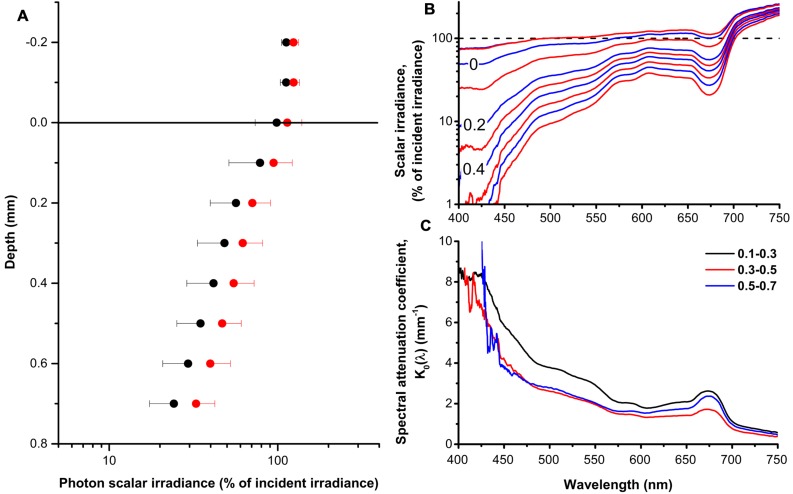
**The coral tissue light field. (A)** Profiles of photon scalar irradiance (PAR, 400–700 nm; black circles) and red light (630–700 nm; red circles) in the polyp tissue of *Montastrea curta* expressed in % of incident downwelling photon irradiance. Note the log scale. Symbols represents means ± 1 SD (*n* = 5); note that for clarity, only minus SD is depicted for PAR and plus SD for red light. **(B)** Profiles of spectral scalar irradiance in the polyp tissue of *M. curta* normalized to the incident downwelling photon irradiance. The dashed black line represents the incident irradiance (100%) and alternating blue and red lines represents measurements in 0.1 mm vertical depth increments from 0.2 mm above the tissue surface and toward the skeleton. Data represent means (*n* = 5). **(C)** Spectral attenuation coefficients, *K*_0_(λ), calculated over 0.2 mm thick tissue zones from the surface toward the skeleton. Lines represent means (*n* = 5).

Both PAR (400–700 nm) and red light (630–700 nm) was attenuated exponentially from the coral tissue surface toward the skeleton (**Figure 1A**), with photon scalar irradiance attenuation coefficients (*K*_0_) of 1.8 mm^-1^ (*R*^2^ = 0.98) and 1.7 mm^-1^ (*R*^2^ = 0.99) for PAR and red light, respectively. Over the coral tissue layer, PAR varied from 107% of incident photon irradiance at the tissue surface to 24% 0.7 mm below the tissue surface, while red light was reduced from 124 to 33% of incident photon irradiance over the same tissue thickness. Scalar irradiance transmission spectra measured in the coral tissue showed characteristic minima and shoulders corresponding to absorption peaks of major zooxanthellate pigments such as Chl *a* (430–440 nm; 675 nm) and Chl *c* (460; 590; 635 nm) (Halldal, 1968; Shibata and Haxo, 1969; Kühl et al., 1995) (**Figure 1B**). In addition, we found indications of host fluorescent pigments that emitted light at longer wavelengths (450–575 nm) when excited with low wavelength blue light (390–410 nm) (Supplementary Figure S2). The host fluorescence partly concealed the spectral signature of the dinoflagellate carotenoid peridinin (490 nm). Scalar irradiance attenuation was higher near the tissue surface (0.1–0.3 mm) and near the skeleton/tissue interface (0.5–0.7 mm) as compared to the middle part of the tissue (0.3–0.5 mm) (**Figure 1C**).

### Photosynthesis and O_2_ Conditions

**FIGURE 2:**
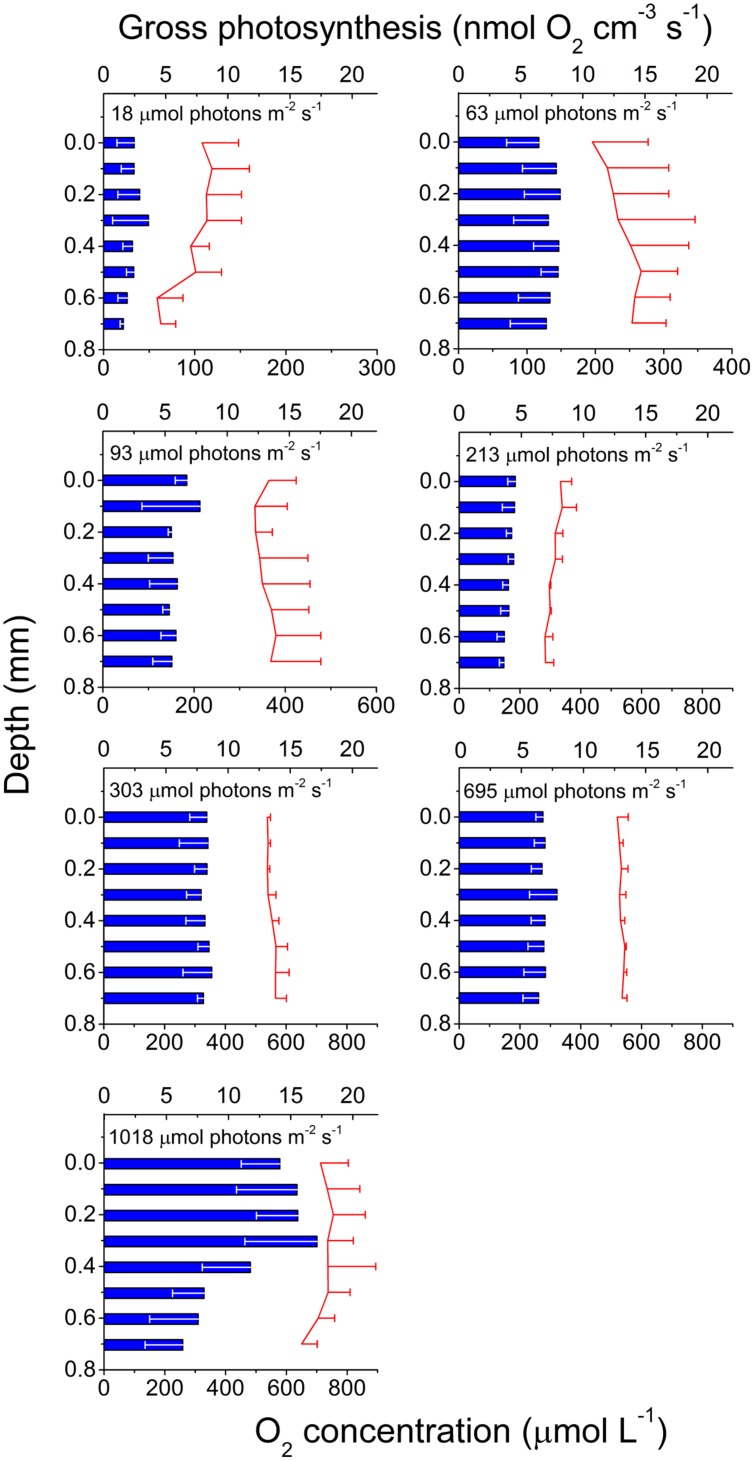
**Photosynthesis in coral tissue**. Depth profiles of gross photosynthesis (nmol O_2_ cm^-3^ s^-1^; blue bars) and O_2_ concentration (μmol l^-1^; red lines; note difference in O_2_ concentration scale between panels) in the polyp tissue of *M. curta* at increasing incident photon irradiance (18, 63, 93, 213, 303, 695, and 1018 μmol photons m^-2^ s^-1^). Data represent means ± 1 SD (*n* = 3). Note that for clarity, only minus SD is shown for gross photosynthesis, and plus SD for O_2_ concentration.

Gross photosynthesis rates generally increased with irradiance and O_2_ production was measured at all tissue depths. The highest gross photosynthesis rate (>17 nmol O_2_ cm^-3^ s^-1^) was measured ∼0.3 mm inside the tissue in the highest light treatment (**Figure 2**). The vertical distribution of production was rather uniform and did not follow the tissue light gradient as would be expected, except for the highest light treatment (1018 μmol photons m^-2^ s^-1^) where production decreased with depth toward the skeleton. Tissue O_2_ concentration ranged between 59 μM in the lowest light treatment (18 μmol photons m^-2^ s^-1^) and 755 μM in the highest light treatment (1018 μmol photons m^-2^ s^-1^) and was uniformly distributed with depth.

### Chlorophyll Fluorescence

**FIGURE 3:**
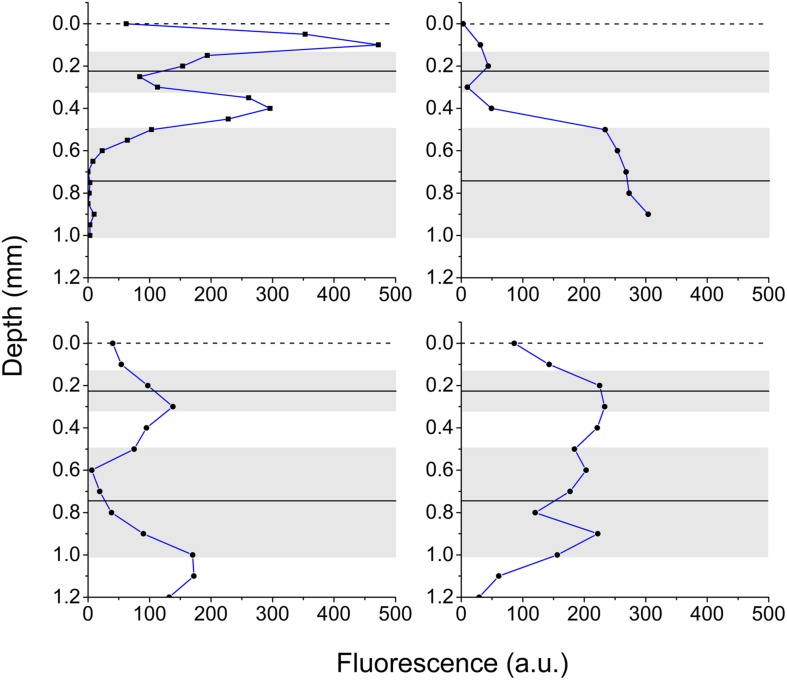
**Chlorophyll fluorescence in coral tissue**. Vertical profiles of Chl *a* fluorescence in the polyp tissue of *M. curta* measured from the surface toward the skeleton. Panels show four replicates demonstrating the heterogeneity of the position of the pigmented *Symbiodinium* layers, in part affected by tissue contraction/relaxation. Solid black lines is the average position of the center of the fluorescent peaks and the gray area depicts ± 1 SD (*n* = 4).

Vertical microprofiles of Chl *a* fluorescence revealed two spatially separated fluorescence bands (**Figure 3**). The zones of enhanced fluorescence were of varying intensity, and the vertical position of both bands differed. An upper peak was detected at 0.23 mm ± 0.10 and a lower peak was detected at 0.75 mm ± 0.27 (means ± SD, *n* = 4). However, depending on the contraction status of the polyp tissue, the bands could be located closer to each other or farther apart. From these measurements, the *Symbiodinium* bands were defined as the zones that exhibited the highest fluorescence (**Figure 3**) and in combination had the highest spectral attenuation coefficient [*K*_0_(λ); **Figure 1C**], and thus we defined the oral band to be located around 0.2 mm and the aboral band around 0.7 mm below the coral tissue surface.

### Photosynthetic Electron Transport of Zooxanthellae *in hospite*

**FIGURE 4:**
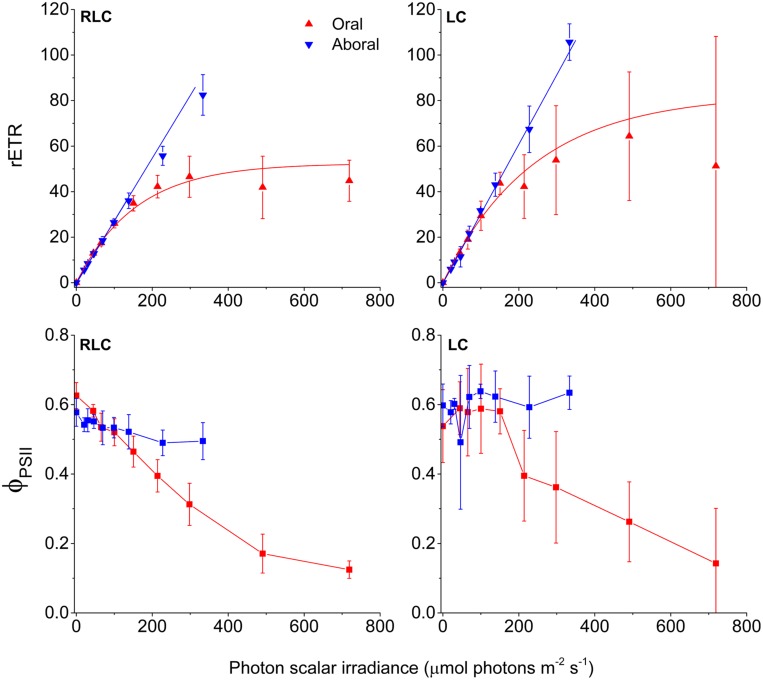
**PSII activity in coral tissue**. Relative electron transport rates through PSII (rETR) **(top)**, and effective quantum yields of PSII photochemistry (ϕ_PSII_) **(bottom)**. Red and blue triangles represent measurements in the oral and aboral *Symbiodinium* bands, respectively. Measurements from left panels were acquired with 10 s acclimation to increasing irradiances (RLC), while the right panels show steady state light curves (LC) using a 5 min acclimation period to each increasing irradiance steps. Data were corrected for the actual scalar irradiance measured at the depth horizons of the defined *Symbiodinium* bands. Curve fits of rETR vs. scalar irradiance were done using either an exponential function (oral tissue layer) (Webb et al., 1974) or a linear curve fit (aboral tissue layer). Data points represent means ± 1 SD (*n* = 4 (RLC); *n* = 3 (LC).

The measured photon scalar irradiance profiles allowed us to estimate the amount of red actinic light available for photosynthesis in different tissue depths, and thereby to relate these values to the variable chlorophyll fluorescence-derived measurements of relative photosynthetic electron transport rates and effective quantum yields of PSII related photochemistry (**Figure 4**). The light available for photosynthesis was 71 and 33% of incident photon irradiance in the oral (0.2 mm) and aboral (0.7 mm) *Symbiodinium* band, respectively (**Figure 1A**).

Effective PSII quantum yields (ϕ_PSII_) and relative rates of electron transport through PSII (rETR) were calculated for the two *Symbiodinium* layers, and were related to the actual photon scalar irradiance of red actinic light (630–700 nm) in each zone (**Figure 4**). ϕ_PSII_ and rETR rates were obtained both from RLC and steady state LC. Both types of photosynthesis vs. irradiance curves showed that the oral *Symbiodinium* layer reached saturation and approached an asymptotic rETR_max_ value of 53 and 83, for RLC and LC respectively. ϕ_PSII_ decreased with increasing irradiance in the oral layer and reached a value <0.2 at the highest light treatment. The aboral *Symbiodinium* layer, however, did not experience sufficient irradiance levels to become saturated, and both RLC and LC measurements showed that rETR continued to increase with irradiance without reaching saturation, with ϕ_PSII_ values in the high light treatment, similar to values in low light (Supplementary Table S1). At the highest irradiance, rETR rates in the aboral layer reached 83 and 106, for the RLC and LC respectively.

The efficiency of light utilization, i.e., the initial slope (α) of the rETR vs. scalar irradiance curves were similar in all treatments, but was generally higher in the oral layer as compared to the aboral layer (Supplementary Table S1). In addition, steady state LC exhibited higher α values than RLC measurements.

The scalar irradiance at the onset of light saturation (E_k_) for the oral layer was calculated as *E_k_ = rETR_max_/α* reaching 156 μmol photons m^-2^ s^-1^ and 239 μmol photons m^-2^ s^-1^ for the RLC and LC measurements, respectively. Because rETR in the aboral *Symbiodinium* layer did not saturate over the investigated irradiance range, it was not possible to calculate the *E*_k_ parameter for this layer.

## Discussion

**FIGURE 5:**
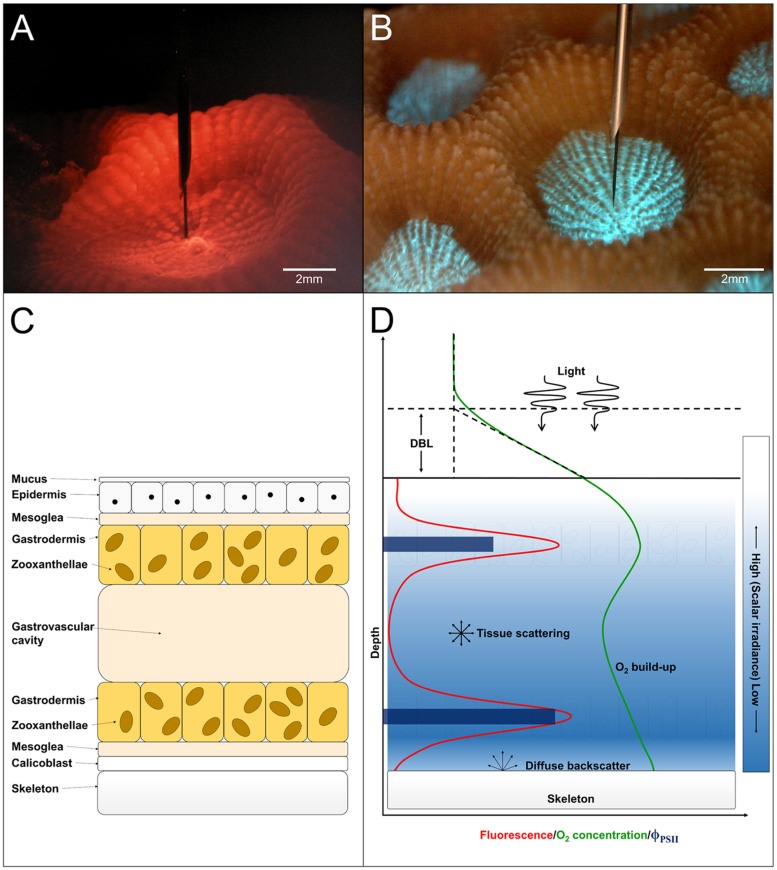
**(A)** Image of a *M. curta* polyp during measurement of scalar irradiance under red light illumination. Scale bar = 2 mm. **(B)** Image of host pigment fluorescence centralized around the mouth in a *M. curta* polyp when illuminated with blue light (390–410 nm). Scale bar = 2 mm. **(C)** Schematic drawing of a tissue cross-section showing anatomical features in coral polyp tissue. From top toward bottom: the mucus layer covering the coral epidermis underneath. Between the two epithelial layers (epiderm, and gastroderm) is the mesoglea. The gastrodermis containing the zooxanthellae surrounds the gastrovascular cavity and underneath the second mesoglea layer is the calicoblastic layer which, excretes the CaCO_3_ and the organic matrix forming the skeleton. **(D)** Conceptual model of the photosynthetic parameters *in hospite* in coral tissue. Light, which is attenuated with depth (blue gradient), drives photosynthesis and O_2_ production in the *Symbiodinium* layers as measured by variable Chl *a* fluorescence (red line). The O_2_ concentration (green line) is a product of photosynthetic production, consumption via respiration of host and symbiont cells and transport by diffusion across the diffusive boundary layer (DBL). The downward flux of O_2_ is spatially restricted by the skeleton, which presents a diffusion barrier enhancing the increase in tissue O_2_ concentration. The skeleton also acts as a barrier for photon flux and light will partly be diffusely backscattered to the tissue. The quantum yield of PSII photochemistry (dark blue bars) was higher in the aboral compared to the oral *Symbiodinium* layer, which gives basis for an equal O_2_ production despite lower light availability.

To the best of our knowledge, we report the first measurements of PSII quantum efficiency and relative electron transport rates (rETR) measured internally in coral tissues, i.e., at the position of the zooxanthellae *in hospite*. Numerous studies have been published on *Symbiodinium* photophysiology in culture, often kept in exponential growth (Iglesias-Prieto and Trench, 1994; Reynolds et al., 2008; Szabó et al., 2014). However, the microenvironmental conditions of *Symbiodinium in hospite* within the coral host tissue differ significantly from conditions in the surrounding water or at the coral-water tissue interface (Kühl et al., 1995; Wangpraseurt et al., 2012; Barott et al., 2015) and the proliferation of symbionts is controlled to some extent by the host (Davy et al., 2012; Cunning and Baker, 2014; Cunning et al., 2015). This study thus provides novel information on the photophysiology of *Symbiodinium* inside coral tissue under natural gradients of chemical parameters and light. Based on our findings combined with existing knowledge, we present a conceptual model on the photophysiology of *Symbiodinium in hospite* (**Figure 5**).

### Scalar Irradiance

Light levels in the coral tissue were similar to values previously found in the same species (Wangpraseurt et al., 2012; Brodersen et al., 2014), and the small differences in attenuation as compared to these studies can probably be ascribed to either intercolonial differences or specific differences in light adaptation as natural light exposure (i.e., sun toward shade colonies) have been shown to be a determinant of tissue light penetration (Ulstrup et al., 2006). The penetration of light is affected by the concentration of light absorbing pigments (Dubinsky et al., 1984), the tissue type (coenosarc or polyp Wangpraseurt et al., 2012) and thickness, which can vary substantially with colony size (Anthony et al., 2002) and over time (Wangpraseurt et al., 2014a).

The coral tissue itself may act as an important determinant of the *Symbiodinium* light microclimate *in hospite* (Wangpraseurt et al., 2012, 2014a,b) in addition to the contribution of diffuse backscattered light of the skeleton to the internal tissue light field (Enriquez et al., 2005; Marcelino et al., 2013). The spectral composition of scalar irradiance changed progressively from the tissue surface toward the skeleton, and our spectral data showed distinct absorption signatures of coral photopigments (Chl *a*, Chl *c* and the carotenoid, peridinin), thus altering the intra tissue spectral light composition with depth. Consequently, e.g., blue light (400–500 nm) was effectively reduced to <10% in the lower tissue layers, while light outside the spectral region of the major photopigments (550–650 nm) only decreased to 32% of the incident irradiance (**Figure 1B**). In addition, we showed a stratification of absorption properties, where the spectral attenuation coefficients [*K*_0_(λ)] were higher in the oral and aboral layers as compared to the central tissue layer (**Figure 1C**). This observation correlates with the vertical distribution of the symbiont biomass as approximated by the vertical Chl *a* fluorescence profiles.

### Photosynthesis and Oxic Environment

The vertical distribution of gross photosynthetic production is typically correlated with the attenuation of scalar irradiance and the distribution of photosynthetic elements (light harvesting biomass), e.g., in biofilms and microbial mats (Kühl et al., 1996; Kühl and Fenchel, 2000). In the investigated coral tissue, production exhibited a relatively uniform vertical distribution and with no apparent correlation with the distribution of scalar irradiance or Chl *a* fluorescence. This apparent mismatch between biomass and production can be a result of, e.g., adaptation of the photosynthetic elements to local quality and quantity of light (Falkowski and Owens, 1980). In higher plants, it has, e.g., been shown that the production along a vertical transect through the leaf did not follow the internal light gradient (Nishio et al., 1993; Sun et al., 1998). Recently gradients of carbon fixation were measured within coral tissues suggesting a different light use efficiency of oral and aboral tissue layers (Wangpraseurt et al., 2015), and microgradients of photosynthetic quantum efficiencies (i.e., mol O_2_ produced per mol photons absorbed) measured in *M. curta* (Brodersen et al., 2014) showed a progressive increase in quantum efficiency with depth. Effectively, this means that aboral tissues, with higher quantum efficiencies, have the ability to produce more O_2_ per mol quanta absorbed than the oral tissue, and thus contribute equally to O_2_ production despite the lower light availability. Photosynthetic O_2_ production increased with increasing photon irradiance and the highest photosynthetic rates were found in the high light treatment. Similarly, the O_2_ concentration was almost constant at all tissue depths, but increased with increasing incident irradiance. Unlike many other photosynthetic systems (e.g., photosynthetic biofilms, macroalgae and other aquatic macrophytes, etc.) coral tissues are spatially constricted by the skeleton which, can act as a diffusion barrier. This has been shown to create a build-up of O_2_ toward the tissue-skeleton interface (Kühl et al., 1995; Wangpraseurt et al., 2012; Brodersen et al., 2014). The rather uniform O_2_ concentration across the coral tissue can be explained in terms of (i) the higher efficiency of photosynthesis in the aboral band of zooxanthellae, (ii) the enhanced scalar irradiance in these lower regions due to back-scattering from the skeleton, and (iii) the fact that the skeleton impedes O_2_ diffusion into the skeleton matrix.

### Quantum Yield and Photosynthesis of Zooxanthellae *in hospite*

The external light field is a poor proxy for the internal light microenvironment experienced by the coral symbionts (Kaniewska et al., 2011). Furthermore, the photosynthetic elements, i.e., *Symbiodinium* cells, in coral polyp tissues are vertically structured as they reside in the gastrodermal tissue layers surrounding the gastrovascular cavity (Fitt and Trench, 1983; Barott et al., 2015) (**Figure 5C**). Thus, to characterize photosynthesis under the conditions experienced by the symbionts inside the tissue of living coral, we measured effective quantum yields of PSII (ϕPSII) and derived relative electron transport rates of PSII photochemistry (rETR), and related them to the actual scalar irradiance at the position of the symbionts.

The symbionts reside in the gastrodermal tissue near the tissue surface and the tissue-skeleton interface (**Figure 5C**). At the coral tissue surface, the photon scalar irradiance is highest and then attenuates exponentially with depth in the tissue. However, the photons reaching the skeleton can also be partly backscattered leading to a photon flux from multiple directions leading to an unexpected level of scalar irradiance in the lower zones (Enriquez et al., 2005; Wangpraseurt et al., 2014a).

The layering of photosynthetic elements in combination with strong gradients of irradiance and spectral composition can cause substantial light-driven stratification of light use efficiency and photosynthesis, even over very small distances (Lichtenberg and Kühl, 2015). The vertical attenuation of light in coral tissue is strongest in the blue region leaving a larger fraction of red light in the lower tissues (Wangpraseurt et al., 2012). In our microscale variable chlorophyll fluorescence measurements, we used red light (630–700 nm; peak wavelength 666 nm) for measuring light, saturating pulses and actinic light driving photosynthesis. Effectively, this could mean that the aboral *Symbiodinium* band were provided with light that they are naturally adapted to, while the oral layer were given a disproportionately large fraction of red light relative to natural spectral distribution in the upper tissue layers.

Recently, PSII absorption cross-sections of *Symbiodinium* (σ_PSII_) were measured, both in culture and *in hospite*, (Szabó et al., 2014) showing a progressive decrease in σ_PSII_ from blue toward red light. However, this was done either by surface measurements of intact corals or in culture with symbionts adapted to culture conditions. We propose that *Symbiodinium in hospite* might display different adaptation to spectral quality depending on their vertical position in the coral tissue. This could explain the observed difference in effective quantum yield and concomitant photosynthetic electron transport in the oral- and aboral symbiont layers (**Figure 4**), where the aboral layer did not saturate, even at the highest experimental irradiance. Similar phenomena have been observed in leaves of terrestrial plants, where a clear difference in absorption of monochromatic blue, green, and red light was observed in the palisade and spongy mesophyll layers (Vogelmann and Han, 2000), and where others have shown that, deep within leaf tissues, green light drives photosynthesis more effectively than red and blue light (Terashima et al., 2009).

In corals, maximum photochemical efficiencies (F_v_/F_m_) in the outer- and inner *Symbiodinium* bands have been estimated by variable chlorophyll fluorescence, albeit by a much more invasive method, i.e., by fracturing the skeleton, and measuring the inner *Symbiodinium* band perpendicular to the surface, demonstrating higher maximum photochemical efficiencies in the inner- relative to the outer *Symbiodinium* layer and with similar initial slopes on the subsaturated part of the rETR vs. irradiance curve (Edmunds et al., 2012). The findings were supported by Brodersen et al. (2014) who showed that the local quantum efficiency (i.e., mol O_2_ produced per mol photons absorbed) increased in deeper lying tissue regions.

Corals are able to optimize light conditions for their symbiotic algae leading to high quantum efficiencies (Dubinsky et al., 1984; Brodersen et al., 2014) and the question of how corals optimize their internal light environment for their photosymbionts has been studied intensively in terms of, e.g., (i) regulation of zooxanthellae pigment density (Falkowski and Dubinsky, 1981), (ii) increased internal light absorption due to backscattering from the skeleton (Enriquez et al., 2005), tissue scattering and light guiding phenomena (Wangpraseurt et al., 2012, 2014a), as well as (iii) wavelength transformation by fluorescent host pigment complexes (Schlichter et al., 1986; Schlichter and Fricke, 1990; Salih et al., 2000; Gilmore et al., 2003). In high-light exposed areas, coral host pigments can absorb radiation in the harmful UV-A and blue range and re-emit photons outside the main peaks in the photosynthetic action spectra (Salih et al., 2000), while in shaded and light limited areas it has been speculated that host pigments can transform radiation outside the photosynthetic action spectra into wavelengths that overlap with the main absorption peaks of Chl *a*, *c* and accessory pigments (Schlichter et al., 1986). In this study we did not quantify host pigment but we found clear evidence for fluorescent host pigments transforming blue light (390–410 nm) into longer wavelengths (460–560 nm) (Supplementary Figure S2).

We performed the first intra-tissue measurements of variable chlorophyll fluorescence in intact corals using a tapered optical fiber with a rounded tip of ∼30 μm; this brings the sampling volume (i.e., spatial resolution) down to approximately the same size around the fiber-tip (Kühl, 2005). Such high resolution measurements are inherently prone to reflect heterogeneities in the organization of phototrophs, i.e., the position of zooxanthellae in the coral tissue, but also enables precise measurements in particular tissue layers. This is in contrast to other fiber- or imaging-based variable chlorophyll fluorescence measurements (see e.g., Schreiber, 2004; Szabó et al., 2014), where a larger surface area is monitored without precise knowledge of the excitation light penetration depth and the relative contributions of different layers to the measured signal; albeit chlorophyll fluorescence from surface layers typically contribute more than layers further away. While correction procedures for variable chlorophyll fluorescence measurements in dense algal cultures have been proposed (Klughammer and Schreiber, 2015), similar corrections in complex stratified tissues such as corals or leaves of higher plants are contrived by their intricate optical properties including the close coupling of scattering and absorption processes affecting light attenuation. In contrast, microfiber-based variable chlorophyll fluorescence analysis can obtain detailed local information on the photosynthetic activity in particular tissue layers under natural light gradients (see e.g., Lichtenberg and Kühl, 2015). Optimally, it requires a sample that does not change anatomical organization as such change might change the relative position of the fiber tip and photosynthetic cells inside the tissue. This was indeed a challenge in the current study in living coral tissue as seen by the large standard deviations in the steady state LC, where the longer acclimation time to increasing irradiance allowed the coral tissue to relax or expand; such tissue change is probably an important regulatory mechanism of the internal light climate (Wangpraseurt et al., 2014a). The position of the symbiont, may thus have changed during measurements of steady state conditions.

Our study of *Symbiodinium* photosynthesis *in hospite* under real tissue light gradients was done under red illumination. The role of spectral composition is undeniably important as it affects processes such as light harvesting (Vogelmann and Han, 2000; Szabó et al., 2014), photoinhibition (Oguchi et al., 2011), respiration (Wangpraseurt et al., 2014c), CO_2_ fixation (Sun et al., 1998), and O_2_ production (Kühl et al., 1995), and we note that the results may differ under white light illumination, although the internal light field in coral tissues is red shifted (Wangpraseurt et al., 2014a).

There is now a need to further investigate the photophysiology of the separate *Symbiodinium* bands at different tissue contraction states (e.g., using a suitable tissue relaxant) to investigate how tissue distribution may be involved in optimizing light utilization of the coral photobionts. In addition, differential adaptations to not only light quantity but spectral composition in the two endosymbiont layers should be studied.

## Author Contributions

ML, AL, and MK designed the research; ML performed the research; ML, AL, and MK analyzed the data; ML wrote the paper with editorial help from AL and MK.

## Conflict of Interest Statement

The authors declare that the research was conducted in the absence of any commercial or financial relationships that could be construed as a potential conflict of interest.
