# Supplementary material for: Photosynthetic Acclimation of Symbiodinium in hospite Depends on Vertical Position in the Tissue of the Scleractinian Coral Montastrea curta
Source: Front Microbiol. 2016 Feb 26;7:230. doi: 10.3389/fmicb.2016.00230 (PMC4768073; doi:10.3389/fmicb.2016.00230)
Supplement: Supplementary file 1 [file Presentation_1.PDF]

Supplementary material

Photosynthetic acclimation of *Symbiodinium in hospite* depends on vertical position in the tissue of the scleractinian coral *Montastrea curta*

Mads Lichtenberg[1*], Anthony W. D. Larkum[2], Michael Kühl[1,2]
[1]Marine Biological Section, Department of Biology, University of Copenhagen, Denmark.
[2]Plant Functional Biology and Climate Change Cluster (C3), University of Technology Sydney, Sydney, NSW, Australia.

*Correspondence:
Mads Lichtenberg,
mads.lichtenberg@bio.ku.dk

Running title: Photosynthetic acclimation of *Symbiodinium in hospite*

Keywords: canopy effects, chlorophyll fluorescence, microsensors, photosynthesis, photo-acclimation, scalar irradiance, zooxanthellae

Supplementary material

Figure S1 shows the spectral composition of the light sources used in this study as measured with a fiber optic scalar irradiance microprobe (Rickelt *et al.*, 2016) connected to a fiber optic spectrometer (USB2000+, Ocean Optics, FL, USA). Light from the fiber optic tungsten halogen lamp (KL2500-LCD, Schott GmbH, Germany) and the red LED ring (Walz GmbH, Germany; Ulstrup *et al.* (2006) was measured as the downwelling scalar irradiance with the fiber optic microprobe positioned over a black, non-reflective light-well. The fiber optic halogen lamp was used for measurements of vertical microprofiles of scalar irradiance in the tissue of *Montastrea curta* and the red LED ring was used for measurements of gross photosynthesis and measurements of effective quantum yield (ϕ_{PSII}) - and relative electron transport rates (rETR) of PSII photochemistry. The scalar irradiance used for correcting ϕ_{PSII} and rETR at the position of the symbionts was integrated from 630-700nm (Figure S1; dashed lines).

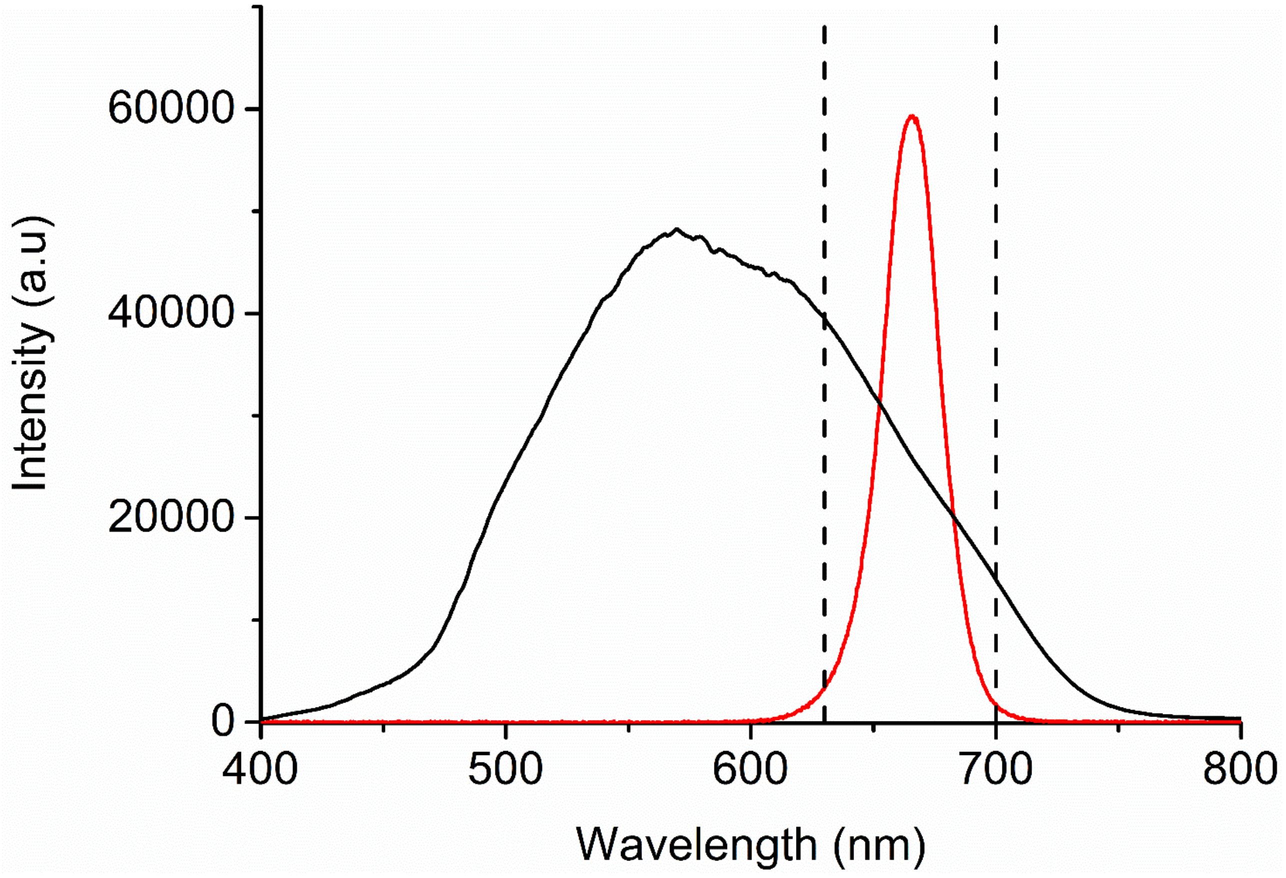

Figure S1: Spectral composition of the fiber-optic halogen lamp (black line) and the red LED ring (red line) used for measurements of gross photosynthesis, effective quantum yield- and relative electron transport rates of PSII photochemistry and the spectral area (630-700nm) (dashed lines) used in the photon scalar irradiance profiles.

Figure S2 shows the distribution and spectral characteristics of host pigment fluorescence measured with a field radiance microprobe when excited with light from a miniature epifluorescence microscope. Field radiance was measured with a field radiance microprobe (tip diameter ~35µm; acceptance half angle ~30°; Kühl and Jørgensen (1992)), connected to a fiber-optic spectrometer (USB2000+, Ocean Optics, Dunedin, FL, USA) controlled by the manufactors software (Spectrasuite, Ocean Optics, Dunedin, FL, USA). The USB microscope (AM4113FVT Dino-Lite, AnMo Electronics Corporation, Taiwan) provided near-UV light (~390-410nm) for excitation of coral host pigments (Figure S2A).

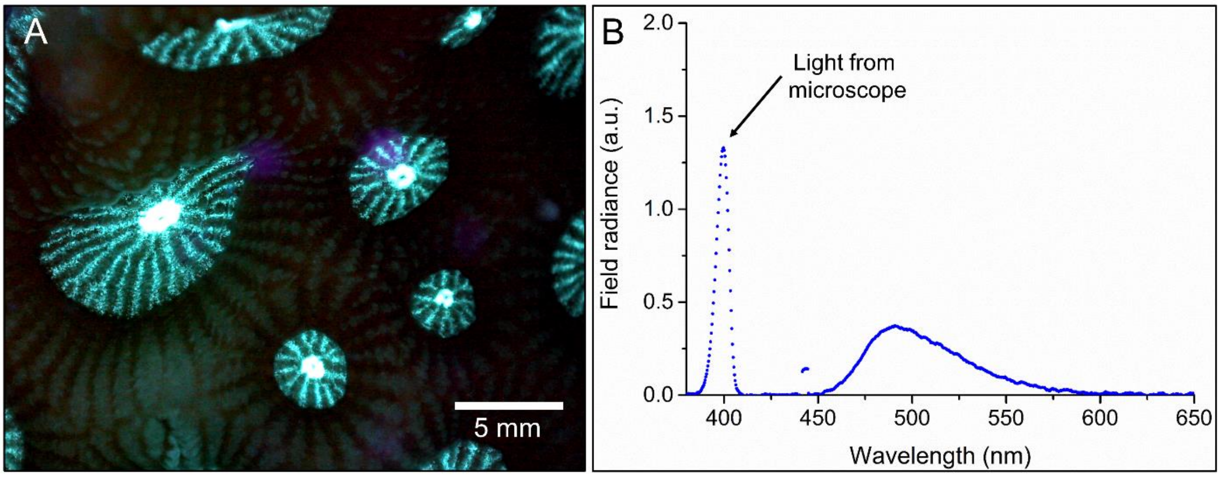

Figure S2: A) Image of host pigment fluorescence from polyps of *Montastrea curta* when illuminated with blue excitation light (390-410nm). **B)** Field radiance measured in the mouth region of a polyp showing the blue excitation light provided by the USB microscope and the host pigment fluorescence at wavelengths >450 nm.

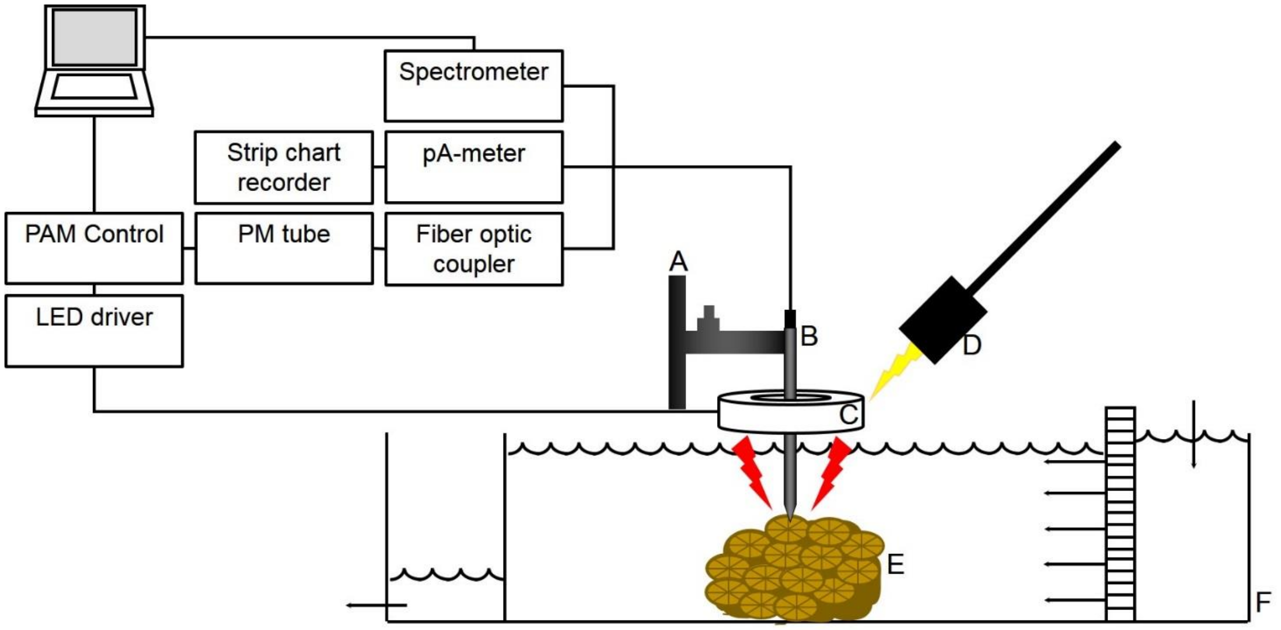

Figure S3: Schematic drawing of the experimental setup. A) a motorized micromanipulator used to move the sensors in vertical steps through the coral tissue, B) microsensors for O_2, scalar irradiance and variable chlorophyll fluorescence, C) LED ring providing external actinic red illumination (used in measurements of O_2 and variable chlorophyll fluorescence), D) fiber optic halogen lamp (used in measurements of scalar irradiance), E) a coral fragment placed in F) the flow chamber ensuring a laminar flow of air-saturated seawater across the coral. O_2 sensor signals were recorded on a strip-chart recorder via a pA meter connected to the microelectrode. The scalar irradiance microprobe was connected to a PC via a fiber-optic spectrometer. The microfiber used to measure variable chlorophyll fluorescence was connected to a PC via the PAM control box, a photo-multiplier tube and a fiber-optic coupler that allowed separation of the excitation light and the detected fluorescence. The red LED ring was controlled on the PC via the PAM control box and the manufacturers software:

Tables

Table 1. Photosynthetic parameters calculated from the variable chlorophyll fluorescence measurements of effective quantum yield (ϕ_{PSII}) and relative electron transport through PSII (rETR) using rapid light curves (RLC) or steady state light curves (LC). Calculated parameters are, light use efficiency (α), maximum rate of electron transport ($rETR_{max}$), the so-called E_k parameter describing the photon irradiance at the onset of photosynthetic saturation and effective quantum yields of PSII related photochemistry (ϕ_{PSII}). $rETR_{max}$ and E_k values for the aboral symbiont band were not calculated as saturation of electron transport was not achieved for the aboral layer.

Parameter	Position	RLC	LC
**α**	Oral	*0.34*	*0.35*
	Aboral	*0.27*	*0.31*
**$rETR_{max}$**	Oral	*52.4*	*82.6*
	Aboral	-	-
**E_k**	Oral	*156.0*	*239.3*
	Aboral	-	-
**ϕ_{PSII}(max)**	Oral	*0.63*	*0.59*
	Aboral	*0.58*	*0.60*
**ϕ_{PSII}(min)**	Oral	*0.13*	*0.14*
	Aboral	*0.50*	*0.49*

References

Kühl M, Jørgensen BB. 1992. Spectral light measurements in microbenthic phototrophic communities with a fiberoptic microprobe coupled to a sensitive diode-array detector. *Limnology and Oceanography* **37**: 1813-1823

Rickelt L, Lichtenberg M, Trampe E, Kühl M. 2016. Fiber-optic probes for small scale measurements of scalar irradiance. *Photochemistry and Photobiology*: doi: 10.1111/php.12560 [Epub ahead of print].

Ulstrup KE, Ralph PJ, Larkum AWD, Kühl M. 2006. Intra-colonial variability in light acclimation of zooxanthellae in coral tissues of *Pocillopora damicornis*. *Marine Biology* **149**: 1325-1335
